# EvoLaps: a web interface to visualize continuous phylogeographic reconstructions

**DOI:** 10.1186/s12859-021-04386-z

**Published:** 2021-09-27

**Authors:** François Chevenet, Denis Fargette, Stéphane Guindon, Anne-Laure Bañuls

**Affiliations:** 1 MIVEGEC, IRD, CNRS, Université de Montpellier, Montpellier, France; 2 IPME, IRD, CIRAD, Université de Montpellier, Montpellier, France; 3 LIRMM, CNRS, Université de Montpellier, Montpellier, France

**Keywords:** Phylogeography, Ancestral character states, Evolutionary scenario, Data visualization

## Abstract

**Background:**

Phylogeographic reconstructions serve as a basis to understand the spread and evolution of pathogens. Visualization of these reconstructions often lead to complex graphical representations which are difficult to interpret.

**Result:**

We present EvoLaps, a user-friendly web interface to visualize phylogeographic reconstructions based on the analysis of latitude/longitude coordinates with various clustering levels. EvoLaps also produces transition diagrams that provide concise and easy to interpret summaries of phylogeographic reconstructions.

**Conclusion:**

The main contribution of EvoLaps is to assemble known numerical and graphical methods/tools into a user-friendly interface dedicated to the visualization and edition of evolutionary scenarios based on continuous phylogeographic reconstructions. EvoLaps is freely usable at www.evolaps.org.

## Background

Phylogeographic reconstructions are used to understand the processes and pace at which organisms colonize their habitat. Nowadays, it is used extensively to study the epidemiology and evolution of pathogens for effective public health measures and surveillance [1–4]. Phylogeographic scenarios come from a root-to-tip reading of a phylogenetic tree annotated with discrete or continuous ancestral character states (locations) [5], computed by Bayesian inference software programs like BEAST [6] or BEAST2 [7]. Visualization of phylogeographic reconstructions may result in complex structures that are difficult to interpret [8].

In this context, we present EvoLaps, a web-based interface dedicated to the visualization and interpretation of phylogeographic reconstructions based on latitude/longitude coordinates. It offers a rich, intuitive and interactive point-and-click interface and drag-and-drop functionalities for clustering locations and customizing phylogeographic visualization.

EvoLaps offers synthetic views of the information conveyed by the data with transition diagrams, allows analyses for data exploration, helps to focus on specific genetic, spatial and/or temporal settings by selecting the appropriate data subsets. EvoLaps also produces ready-to-export figures where the phylogeographic dynamics are superimposed on geographical layers.

## Implementation

### EvoLaps interface

**Fig. 1:**
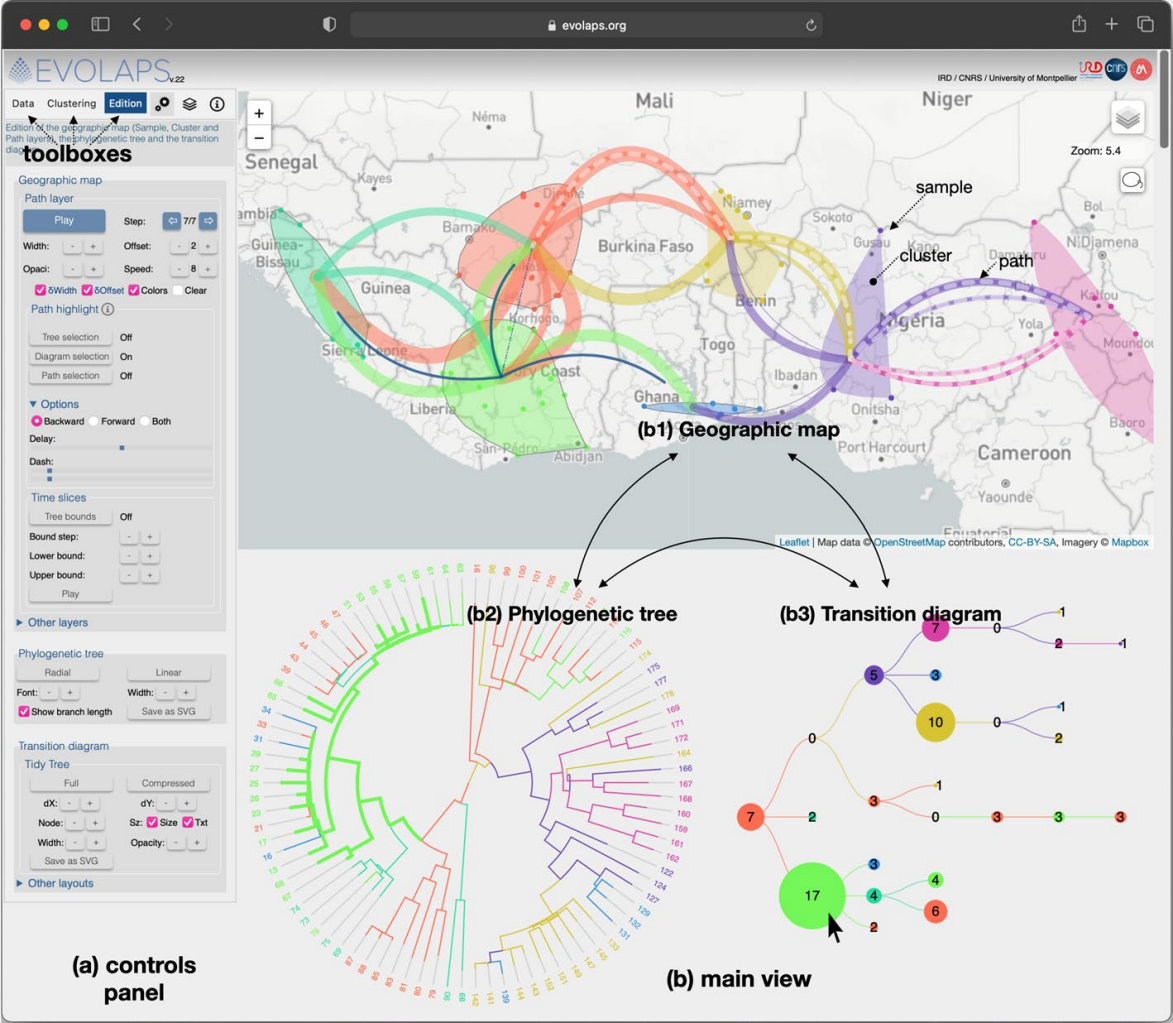
EvoLaps interface. The interface is composed of **a** a left panel displaying three toolboxes, corresponding to the steps of the analysis: ‘data’, ‘clustering’ and ‘edition’; **b** a main view (right panel), subdivided into three components during an analysis: **b1** a geographic map displaying the spatial distribution of the samples, clusters and the resulting paths (spread between the clusters), **b2** the phylogenetic tree displaying the evolutionary relationships among the samples and **b3** a transition diagram summarizing the transitions between the clusters. These three components are inter-connected and they share the same color code based on the current set of clusters defined by the user during the analysis

EvoLaps is a web-based application. The browser side is implemented in HTML 5/JavaScript with several libraries (D3, Leaflet, color2D, jscolor, leaflet-lasso, kmeans, bezier, leaflet.curve, leaflet-ant-path, svg-export), while the server side is based on PHP, Tcl and Python scripts. The EvoLaps interface (Fig. 1) is composed of two panels: a panel displaying three toolboxes on the left side of the interface: ‘Data’, ‘Clustering’ and ‘Edition’ (Fig. 1a) and, on the right side of the interface (Fig. 1b), a main view subdivided into three components during an analysis: a geographic map (Fig. 1b1), the phylogenetic tree (Fig. 1b2) and a transition diagram (Fig. 1b3). The geographic map displays the spatial distribution of samples corresponding to the tips of the phylogenetic tree, the clusters of samples defined by the user during the analysis and the resulting phylogeographic pattern as a set of paths between clusters. This map offers various tile layers in the background (‘light’, ‘satellite’, etc.). The phylogenetic tree displays the evolutionary relationships among the samples. The transition diagram is a synthetic view of the migration history pattern without the geographical constraints (see below). The three components, the geographic map, the phylogenetic tree and the transition diagram, are draggable/zoomable and are inter-connected. They share the same color code based on clusters of geographic locations, and act as interfaces to focus and highlight data subsets on every other component. For instance, a clade selection on the phylogenetic tree highlights the underlying subtree and the corresponding samples on the geographic map, this selection can be used to define a cluster ('cluster' toolbox) or to highlight local geographic paths ('edition' toolbox). A node selection on the transition diagram highlights the related subtree(s) on the phylogenetic tree and the local paths on the geographic map.

### EvoLaps problem solving process

**Fig. 2:**
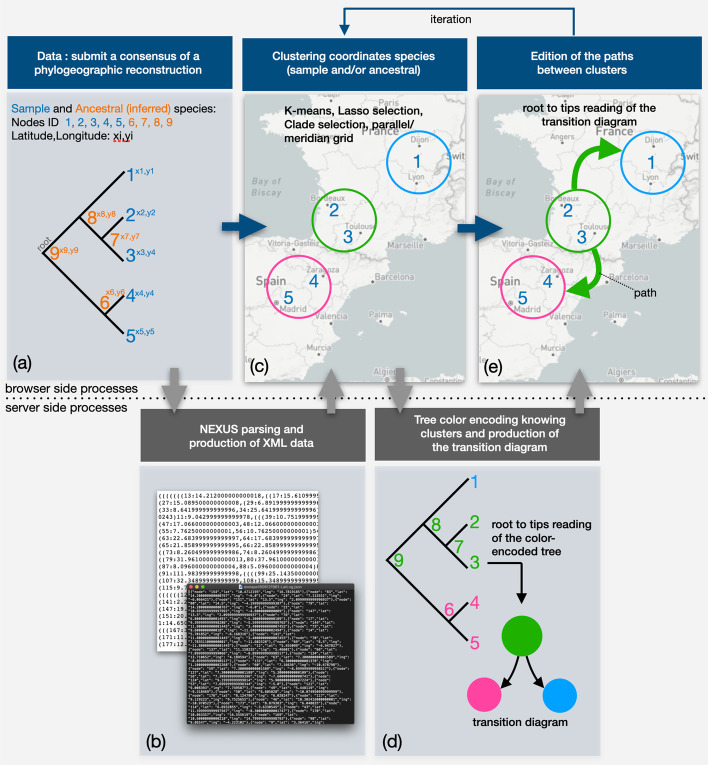
The successive steps of an EvoLaps analysis: **a** first step of the analysis: submission on the fly of a consensus of a phylogeographic reconstruction (NEXUS format), including a newick string for the phylogenetic tree and the latitude/longitude coordinates for each node of the tree (sample and ancestral nodes). **b** server side: the submitted file is parsed to extract and produce JSON files for the tree and the coordinates which are returned to display the tree and the samples on the geographic map; **c** second step of the analysis: clustering of the sample and/or ancestral locations on the geographic map with the help of several methods: K-means algorithm and/or lasso selection and/or clade selection or meridian/parallel grid, clusters are submitted to the server and **d** given a chosen color scale, the tree is color-encoded, the transition diagram is computed in a top-down reading of the color-encoded tree, and is displayed; **e** step three of the analysis: edition of the paths on the geographic map in a top-down reading of the transition diagram. The analysis can be iterated gradually from step two to refine the phylogeographic pattern

An EvoLaps analysis unfolds into three steps: data importation, clustering and edition (Fig. 2):

- The first step (Fig. 2a) corresponds to the importation of data from a third-party software that generates a consensus of a phylogeographic analysis. Input data is submitted on the fly, it must contain a rooted tree (NEXUS format) with samples and ancestral (consensus) latitude/longitude coordinates for the tips (samples) and the internal nodes of the tree (ancestral species), respectively. This file may be generated using TreeAnnotator from the BEAST software package [7], following a Bayesian phylogenetic analysis. On the server side (Fig. 2b), the input file is parsed to extract and save the newick string and the latitude/longitude coordinates of each node under a XML format. Then, back to the browser side, the tree is displayed and the geographic map is updated to display the locations of each tip of the tree;
- Clustering is the second step (Fig. 2c) of the analysis. It consists in gathering the latitude/longitude coordinates of every individual location into geographical areas. Several methods are available to define clusters of locations (see below). Clusters are color-encoded using a linear color scale (mono or polychromatic) or a 2D matrix color scale which is an efficient way to set progressive color changes between close clusters. The cluster/color list is then sent to the server (Fig. 2d). The tree is color-encoded and is read from its root to its tips to compute a transition diagram;
- The third step of the analysis, edition, corresponds to the visualization of the phylogeographic reconstruction (Fig. 2e). The transition diagram is read from its root to its tips: each transition is projected on the map as a path between two clusters. The result is a phylogeographic scenario anchored to the clusters. This scenario is displayed step by step, manually (backward/forward buttons) or automatically (animation with adjustable speed). The phylogeographic pattern can be edited (size and curvature of paths), highlighted (visualization of transition suites) and restricted, thanks to dynamic time slices superimposed on the tree.

The analysis can be iterated from the clustering step. A session starts with a small number of clusters, and the clustering can then be refined on the fly, with subdivisions of one or several of the previous clusters into smaller ones up to a satisfying output.

### EvoLaps clustering mode

**Fig. 3:**
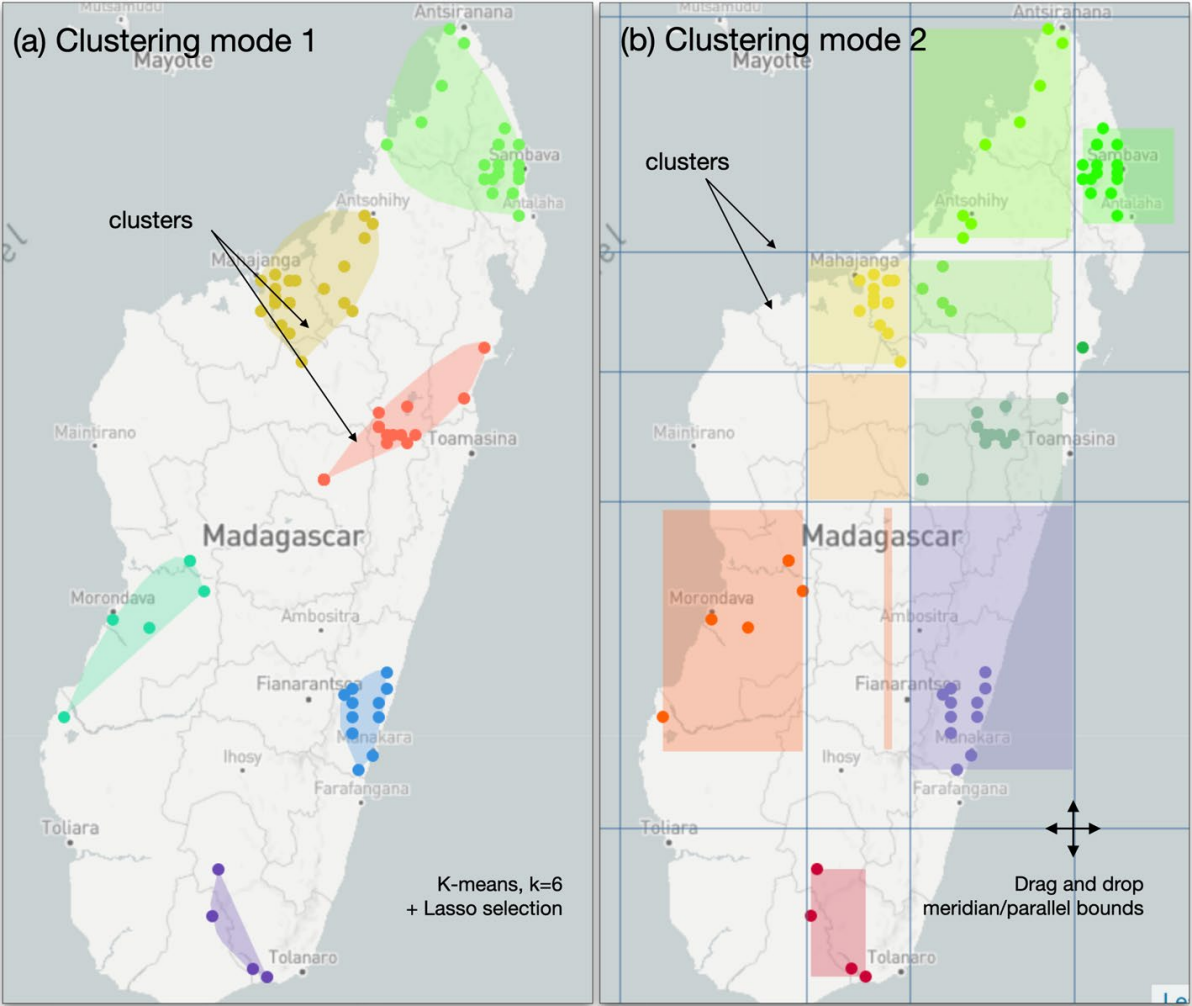
The two clustering modes of EvoLaps. **a** Clustering mode 1, geographic map with the ‘Light’ layer, K-means = 6 and lasso selection. Clusters are represented as smooth polygons. The phylogeographic pattern is anchored on clusters defined with sample locations only. Ancestral locations are bound to their nearest cluster. **b** Clustering mode 2, geographic map with the ‘Light’ layer, drag and drop of latitude and longitude bounds of a meridian/parallel grid. Clusters are represented as bounding boxes of locations containing sample and/or ancestral (computed) locations. Ancestral locations are not shown on the map. The phylogeographic pattern is anchored to the clusters defined on latitude/longitude areas containing either samples and/or ancestral locations

EvoLaps proposes two modes of clustering of coordinates of samples and/or ancestral lineages (Fig. 3).

- **Clustering mode 1** The first mode of clustering does not consider ancestral locations in the definition of the clusters. The user defines clusters of sample locations on the geographic map at a given spatial scale, with the help of methods and selection tools, such as K-means clustering and/or manual lasso selections on the map and/or clade selections from the tree. The K-means algorithm [9] requires (a users’s setting) an initial K number of seeds (cluster centroids) randomly generated within the boundaries of sample locations. Then, iteratively until no change, sample locations are assigned to their closest centroid based on the Euclidean distance and centroids are updated. Clusters are displayed on the map as a list of smoothed polygons containing one or more sample locations (Fig. 3a).
- **Clustering mode 2** The second clustering mode uses a dynamic grid of latitude/longitude bounds to partition the space at a given density and scale, and each bound can be dragged and dropped to produce a more relevant space division. The grid of latitude/longitude bounds subdivides the whole space into regions. If a region contains one or more ancestral and/or sample locations, it contains a cluster. Ancestral locations are thus taken into account in clustering mode 2. Clusters are then displayed on the map as bounding boxes of their locations (Fig. 3b). This clustering mode may also be used with a K-means clustering. In this case, clusters are identified considering sampled locations only, then minimum and maximum of latitude and longitude coordinates of each cluster are used to position bounds of meridian/parallel.

### Phylogenetic tree color-encoding

The phylogenetic tree is color-encoded when a list of clusters and their associated colors is established. Each ancestral location in the tree is associated to one cluster with its specific color, which is used to color the internal node. If clustering mode 1 is used, ancestral locations are associated to the closest cluster (the shortest distance from the ancestral location to the center of clusters). If clustering mode 2 is chosen, ancestral locations are naturally linked to one of the clusters defined by the latitude/longitude grid.

### The transition diagram

**Fig. 4:**
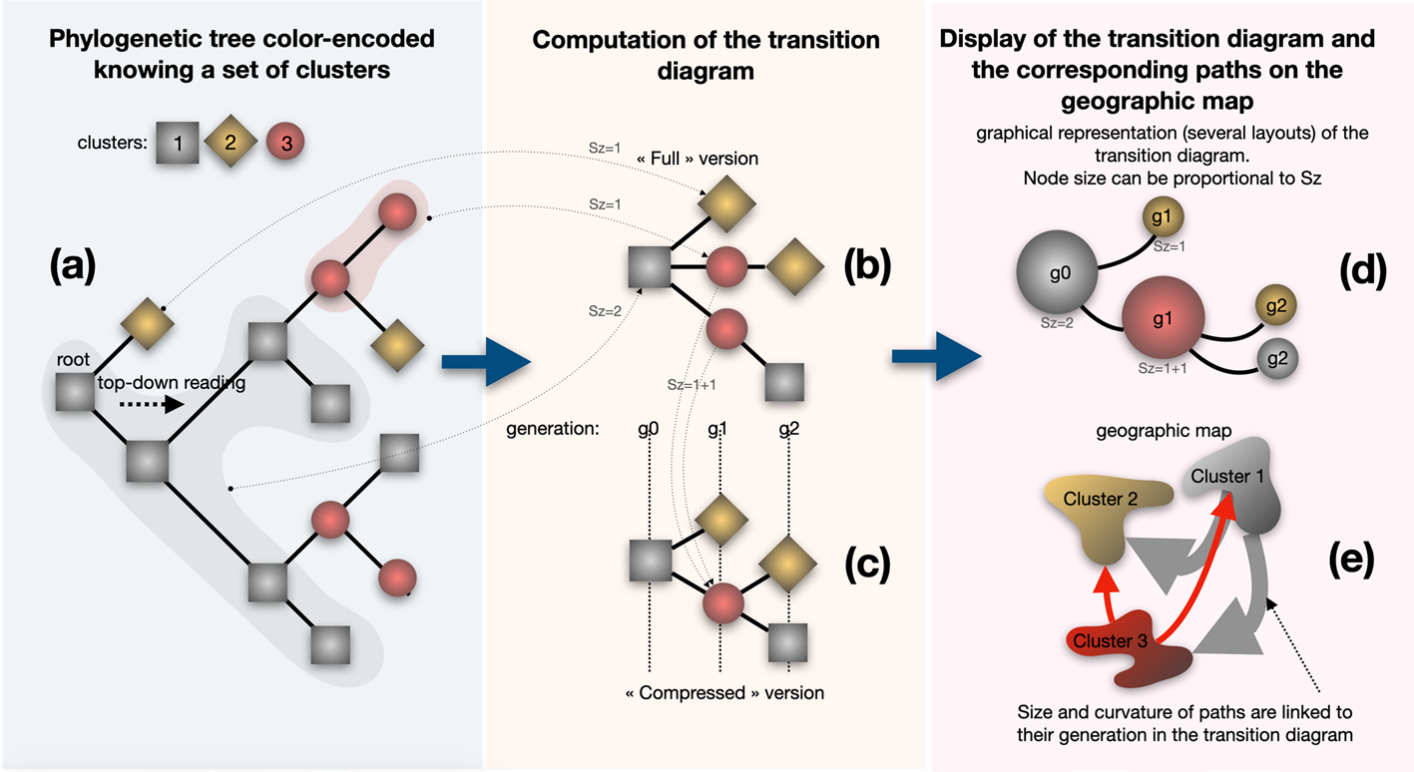
EvoLaps transition diagram. **a** Phylogenetic tree color encoded given a set of clusters: locations (latitude/longitude) of each node (ancestral or sample) of the phylogenetic tree are linked to one cluster/color defined by the user during the analysis (gray squares, red circles and gold diamonds), **b** transition diagram, ‘straight’ version. A node is created in the transition diagram for each transition observed in a top-down reading of the phylogenetic tree (with the exception of the root). **c** Transition diagram, ‘compressed’ version. The ‘compressed’ version of the transition diagram displays similar transitions when they share the same father node in the ‘straight’ version of the transition diagram, **d** tree-like graphical representation of the transition diagram, several layouts, nodes size may reflects the Sz criterion: the number of tips being in the same geographic cluster along the evolutionary path from a transition to its tips. Sz values are added in the compressed version of the transition diagram for the nodes sharing a geographic cluster at the same generation (red node), **e** top-down reading of the transition diagram to produce paths between clusters on the geographic map. Size and curvature of the paths may reflect the generation of the transition

A transition is defined as an inferred migration across a pair of geographical clusters between subsequent nodes of the tree in a top-down reading, from the root to the tips in a recursive process (Fig. 4a). A default diagram starts with a node corresponding to the ancestral root state *i*. The state *i* is the associated cluster knowing the ancestral latitude/longitude linked to the tree node. As in the tree color encoding process, if the first clustering mode was chosen at the clustering step, each ancestral lineage is associated to the cluster that minimizes the distance between the coordinates of the lineage of interest and the center of the cluster examined. Otherwise, if the second clustering mode was chosen at the clustering step, the cluster of the ancestral lineage is directly determined by its coordinates through the parallel/meridian grid. A node is inserted in the transition diagram when a cluster transition *i*—> *j* is observed until the tips are reached (Fig. 4b). A compressed version of the diagram is available by collapsing identical transitions having the same ancestor in the default version (Fig. 4c). The transition diagram is represented as a multi-furcating tree-like representation, summarizing the series of transition that took place during the course of evolution. It gives a synthetic view of a phylogeographic pattern without the geographical constraints (Fig. 4d). Several graphical layouts are available (tidy tree, force-directed graphs, etc.). Nodes sizes can be equal, or proportional to the Sz criterion, which is the count of descendants being in the same geographic cluster along the evolutionary path from a root node linked to a transition, to its tips. In case of a compressed version of the transition diagram, Sz values are added for the nodes sharing a geographic cluster at the same generation. For more details related to the transition diagram and the Sz criterion, we refer the reader to [10–12]. The transition diagram is then read by generation step from its root to its tips to produce paths between clusters on the geographic map (Fig. 4e).

## Results

We reexamined a published study related to the epidemiological history of the *Rice yellow mottle* virus (RYMV) in West Africa [13]. RYMV is one of the tropical plant virus diseases with a high socio-economic impact in Africa. A thorough understanding of RYMV evolution and dispersal is critical to control the viral spread in tropical areas that heavily rely on agriculture for subsistence. Our analysis is composed of two sessions based on the two clustering modes implemented in EvoLaps.

### Study of RYMV in West Africa with a progressive clustering of sampled locations

**Fig. 5:**
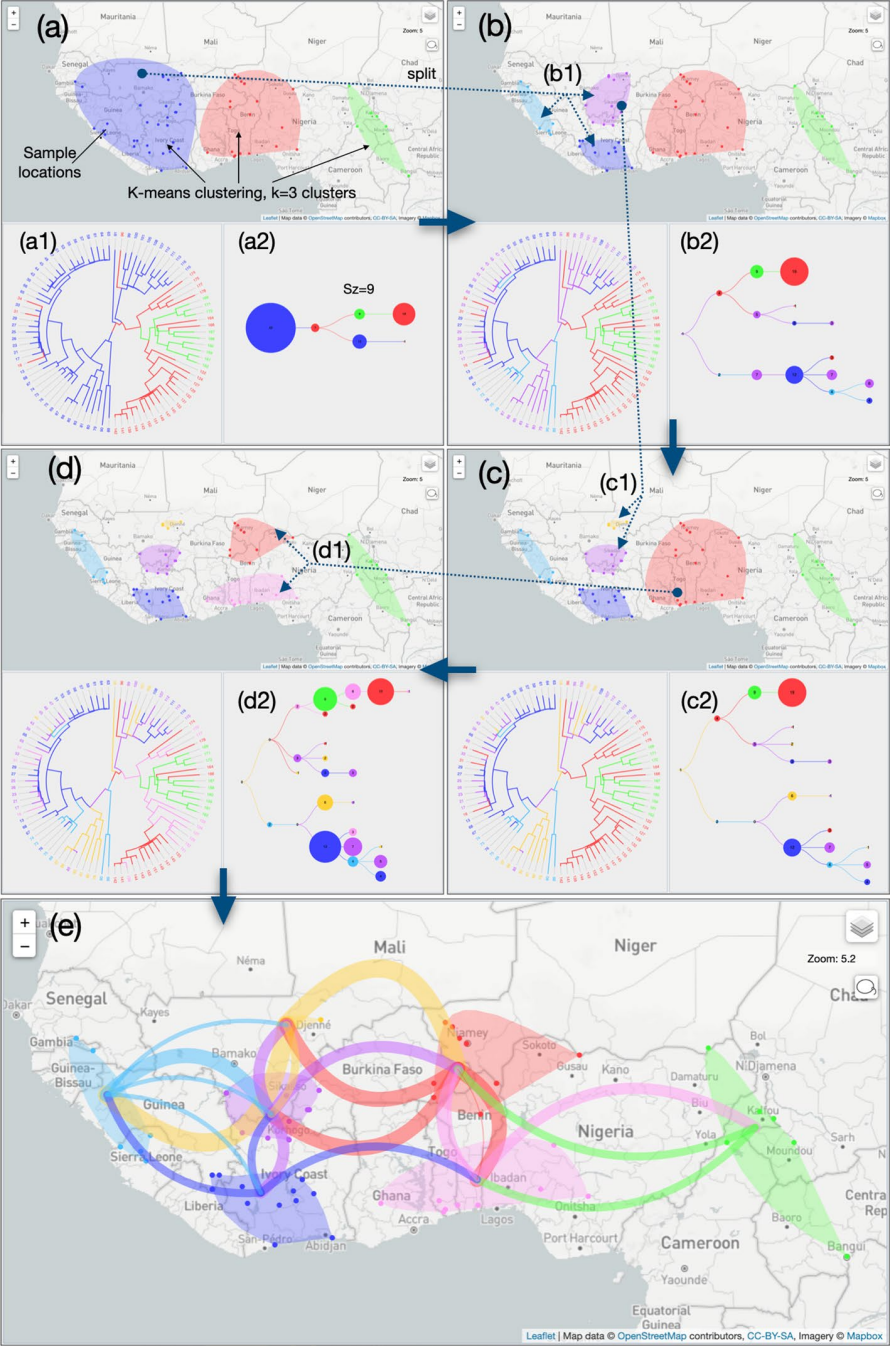
Analysis of the phylogeography of *Rice yellow mottle* virus in West Africa, data from [13]. Screenshots showing the geographic map, the phylogenetic tree and the transition diagram (compressed version) for each step of a progressive clustering mode 1 process, **a** clustering step 1: **a1** K-means clustering (default clustering mode 1) with k = 3 (default number of clusters), ‘Sinbow’ 1D color scale, **a2** the transition diagram (with Sz criterion) shows a ‘blue’ origin of the phylogeographic scenario, **b** clustering step 2: **b1** lasso selection to split the previous blue cluster into three smaller clusters: ‘purple’, ‘light blue’ and ‘blue’ clusters, **b2** the transition diagram specifies a ‘purple’ origin of the scenario, **c** clustering step 3: **c1** lasso selection allows to split the ‘purple’ cluster into two sub-clusters: ‘orange’ and ‘purple’ clusters, **c2** the ‘orange’ cluster is the origin of the scenario, **d** clustering step 4: **d1** the ‘red’ cluster is subdivided into two new clusters by lasso selection to complete the analysis (‘orange’ and ‘pink’ clusters) **d2** last transition diagram, **e** paths between clusters on the geographic map corresponding to the last transition diagram

Our first analysis is based on a progressive clustering of sampled locations (clustering mode 1, Fig. 5). We start with a K-means clustering with the default number of three clusters with the default 1D ‘Sinbow’ color scale (Fig. 5a). The geographic map displays the resulting clusters as smoothed polygons distributed along a West–East transect: ‘blue’, ‘red’ and ‘green’ clusters. Sampled locations are represented as color-encoded dots among their membership cluster. The phylogenetic tree is also color-encoded according to this clustering: ancestral locations associated to the internal nodes of the tree are linked to the closest existing cluster/color (Fig. 5a1). The corresponding compressed version of the transition diagram summarizes the cluster/color transitions from a root-to tips reading of the phylogenetic tree (Fig. 5a2). It shows a West to East dispersal of RYMV. The dispersion spreads from the ‘blue’ to the ‘red’ cluster, then from the ‘red’ to the ‘green’ cluster (with several ‘red’/‘blue’ and ‘green’/‘red’ exchanges). To shed light on the origin of the spread, we go back to the clustering step and split the ‘blue’ cluster into three smaller ones with a lasso selection in three directions (Fig. 5b): West (a ‘light blue’ color is selected manually with the color picker), Northeast (‘purple’ cluster) and South East (‘blue’ cluster) (Fig. 5b1). The phylogenetic tree color code is updated and a new transition diagram is computed, specifying a ‘purple’ origin of the RYMV phylogeographic scenario (Fig. 5b2), now spreading West (‘light blue’) and East (‘red’), the ‘South’ region (‘blue’ cluster) being contaminated later. A third clustering step (Fig. 5c) with a lasso sub-division of the ‘purple’ cluster (Fig. 5c1) pinpoints the origin of the epidemic from the ‘orange’ cluster (Fig. 5c2). To detail the last part of the scenario in the East regions, a last lasso selection is used to split the ‘red’ cluster into a North and a South cluster (‘red’ and ‘pink’ clusters respectively, Fig. 5d1). The ‘green’ cluster in North Cameroon appears to be contaminated from the ‘pink’ cluster. The concluding transition diagram (Fig. 5d2) is used to draw the paths between clusters on the geographic map. Sizes and curvatures of paths are set as a function of their generation in the transition diagram for a more readable output (Fig. 5e). This first EvoLaps session outputs the following final result: the phylogeographic scenario starts from the ‘Djenné’ region in Mali to the west of Guinea and to the east of Burkina Faso (with reference to the geographical center of clusters). From Guinea, it spreads to the south of Mali (south of the ‘Sikasso’ region), then to Ivory Coast. From the east of Burkina Faso, it first spreads south (Togo, Benin), then east (Nigeria/North Cameroon).

### Study of RYMV in West Africa with a dynamic meridian/parallel grid

**Fig. 6:**
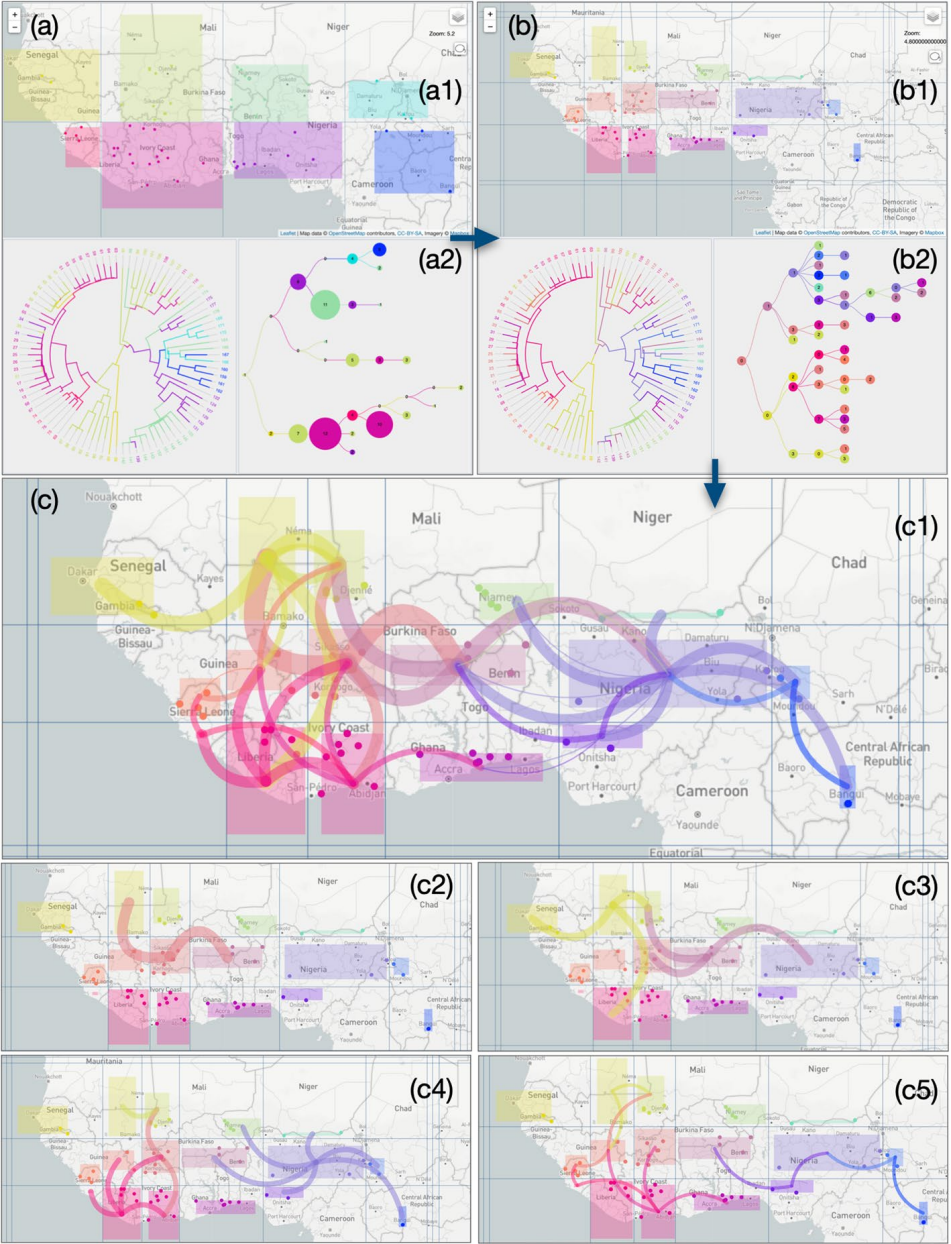
Analysis of the phylogeography of *Rice yellow mottle* virus in West Africa, data from [13]. Screenshots showing the geographic map, the phylogenetic tree and the transition diagram (compressed version) for each step of a progressive clustering mode 2 process. **a** clustering step 1: **a1** default density of the meridian/parallel grid, 2D color scale (‘Steiger’ scale). Eight clusters are displayed as bounding boxes of samples and/or ancestral locations, **a2** the transition diagram (with Sz criterion) shows a ‘pale green’ origin of the phylogeographic scenario, **b** clustering step 2: **b1** higher density and drag and drop of meridian/parallel bounds, **b2** the transition diagram (without Sz criterion) specifies a ‘pale red’ origin of the phylogeographic scenario, **c** paths between clusters based on the last transition diagram, **c1** full scenario, **c2–5** first four steps of the scenario

The second EvoLaps session in the study of RYMV in West Africa is based on a meridian/parallel grid for clustering samples and ancestral locations (clustering mode 2, Fig. 6). It starts with a default density of latitude/longitude bounds superimposed to the geographic map and resulting in a North/South latitude bound crossing five East/West longitude bounds. This partition divides the geographic map into eight regions of equal sizes (Fig. 6a1). Each region is associated to a color from a 2D color scale (default ‘Steiger’ scale based on the center of the region) and is a potential cluster. This geographic partition is submitted, and a region is identified as a cluster if it contains one or more sampled and/or ancestral locations. If that is the case, clusters are represented as a color-encoded bounding box based on the minimum/maximum coordinates (latitude and longitude) of its locations (ancestral and/or sampled). Sampled locations are also displayed as colored dots among their membership cluster/region. The phylogenetic tree is color-encoded: internal nodes of the tree are associated to ancestral locations themselves linked to one of the eight regions/colors. The transition diagram is then computed and displayed (compressed version, Fig. 6a2). The dispersion appears to start from the ‘light green’ cluster to the ‘yellow’ (West) and ‘pink’ (South) clusters. From this ‘pink’ cluster a propagation to the east is identified with the sequence ‘pink’ → ‘purple’ → ‘blue’, with a north dispersion at each step (‘purple' to ‘green’, ‘blue’ to ‘light blue’). A higher grid density of meridian/parallel is then used with a drag and drop of longitude bounds to detail regions that have relatively higher rice production. The resulting transition diagram (Fig. 6b2) is projected on the geographic map with sizes and curvatures of paths in terms of generation in the transition diagram (Fig. 6c). The resulting beam of paths is more complex than those obtained previously (Fig. 6c1). For a more readable output, the phylogeographic scenario is decomposed generation by generation into the first four successive steps (generation steps from the transition map) (Fig. 6c2–5). Without going into details, the phylogeographic scenario starts from the ‘light red’ cluster in the Sikasso/Korhogo region, then it spreads, on the one hand, to the north west (‘light yellow’ cluster) followed by a West migration to Senegal and on the other hand, to the East regions: first to the north of Togo, then Nigeria (‘purple’ cluster). A radiation is then observed from this last cluster.

A short comparative analysis of the two studies of RYMV in West Africa allows us to identify the main expansion dynamics: an initial spread from the region between Djenné (Mali) and Korhogo (Ivory Coast) to the Western (Senegal) and Eastern directions (Benin). These outputs are close to the original study. The K-means method followed by successive lasso selections is a powerful approach to adapt the cluster definition to geographical constraints such as countries or routes of transmission such as rivers, but the phylogeographic scenario is restricted to clusters based on sampled locations. The meridian/parallel grid is straightforward in the definition of rectangular regions which is less adequate to adapt the cluster definition to geographic constraints. The density of latitude and longitude bounds can be increased for a higher resolution but often results in more complex scenarios that are difficult to interpret. However, ancestral locations are considered, which is more accurate to study the evolutionary process. Nevertheless, special attention should be paid to the early stages of the evolutionary scenario, near the root of the phylogenetic tree where ancestral locations may be prone to artifacts of computation.

## Conclusion

Thanks to the continuous latitude/longitude coordinates associated to a dynamic clustering process, EvoLaps facilitates the search of phylogeographic patterns based on various aggregation levels of locations. The thinner the clustering, possibly restricted to a specific area, the more detailed the corresponding phylogeographic pattern is. The clustering process integrates several methods (K-means, lasso selection, clade selection, drag and drop of bounds of a meridian/parallel grid) allowing to consider or not the inferred ancestral locations in the cluster list definition. One possible improvement of the clustering step would be the display of ancestral locations on the geographic map and their inclusion in the clustering process using lasso selections. The possibility to save/load a clustering to re-start or share an analysis is in our specifications. The transition diagram is a basic component of the EvoLaps interface. It summarizes the view of the phylogeographic changes and acts as an interface between the tree and the geographical map displaying complex patterns. Future steps in the computation of the transition diagram will consider (1) branch lengths of the phylogenetic tree so as to link the phylogeographic patterns to time with several rooted layouts, (2) simplification of the diagram such as collapsing sequences of transitions and thus summarize multiple exchanges between areas (e.g., transition nodes ‘a-b-a-b’ to a single node ‘a/b’). Given the uncertainty related to the computation of ancestral sequences and character states, it may be of interest to integrate comparative methods/tools to display the corresponding information and highlight potential discrepancies of different outputs (see, for instance, [12]). Also, a challenge will be the inclusion of other sets of ancestral character states, such as drug resistance, to study epidemiological phylodynamics.

## Data Availability

Project name: EvoLaps. Project home page: http://www.evolaps.org/. Operating system(s): Platform independent. Programming language: EvoLaps client side is based on JavaScript with D3.js and Leaflet libraries, while the server side is based on PHP, Tcl and Python scripts. Other requirements: none. License: GNU GPL. Any restrictions to use by non-academics: none. EvoLaps source code and the datasets used in this study are available on http://www.evolaps.org/.
